# Abstracts and Gleanings

**Published:** 1884-01-20

**Authors:** 


### ABSTRACTS AND GLEANINGS.

**Maximum of Carbolic Acid in Antiseptic Gauze.**—Dr. Rupprecht, chief surgeon at the Diakonissen-Anstalt in Dresden, writes to the editor of the Pharmaceutische Centralhalle as follows:

"The results of the last analysis of the commercial Lister's gauze—5·9 per cent. of carbolic acid—affords me an opportunity to ask that you publish my views regarding the highest safe limit of carbolic acid in such dressings. Arnold's as well as Kahnemann's carbolic gauze contains, as you have yourself found, about 6 per cent. of the acid.

If this gauze is applied over expensive surfaces (as a whole leg, or the abdomen) in the larger operations, and covered with impermeable tissue, adults can be rendered seriously ill by the absorption of carbolic acid through the skin; with children up to about five years of age it may be fatal. I perform over 400 operations every year, never wash out a wound with carbolic acid without rinsing afterwards with salicylic acid, and cause the urine of all persons operated upon and dressed to any extent with carbolized gauze, to be tested for carbolic acid (by means of a solution of chloride of barium). For years I have never seen a case of carbolic poisoning, as long as I used a 3·5 per cent. gauze (for children one per cent.), prepared either by myself or specially prepared for me. My stock of gauze had recently run out, the new supply of 3 per cent. gauze had not arrived, and I was compelled to use, during several days, the gauze made by Kahnemann. Although every other step of treatment was the same as in former cases, three of the operated persons nevertheless developed symptoms of carbolic poisoning (vomiting, collapse, cessation of precipitate by barium chloride in the urine), and a child of 5 years of age succumbed. Running the risk of reiterating what should be well known, I beg you will state the following propositions, for which I ask the largest possible circulation:

1. A 3 per cent. gauze (for children 1 per cent.) is amply strong enough to insure an aseptic condition of wounds.

2. A 6 per cent. gauze, employed for larger dressings, may produce death by absorption through the skin; this may easily occur in children.

3. Manufacturers of carbolic gauze would do well to state upon the label the percentage of carbolic acid contained in the product."—*Sleeman's Chemical Circular.*

**Insomnia**—*Croton-Chloral Hydrate.*—On the recommendation of Dr. Collier, of Gorleston, I tried croton-chloral hydrate in doses of from five to ten grains, combined with chloral hydrate in thirty-grain doses; and, on account of the cardiac debility, I added drachm-doses of spirit of ether. This mixture produced sleep more effectually than anything else I have prescribed.—Dr. J. Ryley, Yarmouth, *British Med. Jour.*

**Dropsy.**—*Concentrated Solution of Saline Cathartics.*—A concentrated solution of a saline cathartic ought to prove of considerable service in certain cases of dropsy, where, owing to the great accumulation of transuded serum in vital parts and elsewhere, there is imminent danger to life and an urgent need for an immediate and active removal of a portion of the transuded fluid. In such cases the value of saline and other active cathartics has long been appreciated, but I am not aware that use has been made of the more powerful action of a concentrated saline cathartic. It removes the dropsical fluid by two channels: by the intestines and by the kidneys. No other purgative has this double action. It is questionable, also, if any other purgative acts so rapidly in reducing the fluids of the blood. This is of the greatest importance in certain critical cases of dropsy. It is almost perfectly certain that no other purgative excites intestinal secretion so powerfully, and at the same time produces so little irritation of the intestinal mucous membrane and so little disturbance of the body generally. This is an additional recommendation for the employment of the concentrated saline. The diluted salt, the form in which it is always given, has practically, in so far as it affects dropsical fluids, the action only of a diuretic. Based on these considerations, I have made several trials of the concentrated salt in suitable cases of dropsy, and in most of them with very satisfactory results. In a boy aged 10, I prescribed three-quarters of an ounce of sulphate of magnesia dissolved in two tablespoonfuls of water, no water to be given afterward. The result exceeded my expectation. When I called next evening, the patient was lying quietly sleeping in his bed. The nausea was greatly diminished, and the dyspnœa had almost entirely gone, and his breathing was much slower. The pulse was also less rapid, and the pained, anxious expression of his face had vanished. His mother told me that she had given him the salt as I had directed, and that in less than an hour afterward the purgative action of the salt manifested itself, and there were repeated evacuations in the course of the next few hours; on each occasion the water seemed to "gush" from him, and he passed an unusually large quantity of urine.—Dr. Mathew Hay, p. 67, *Braithwaite's Retrospect.*

**Bowditch's Formula for Irregular Heart.**—In a discussion upon heart-disease before the Boston Society for Medical Improvement, Professor Bowditch said that he had found the following formula of great service in relieving even the most serious cardiac affections. He had used it for the last twenty-five years:

℞. Pulv. digitalis.............................grs. x,
Pulv. colchici sem.......................grs. xx,
Sodii bicarbonatis.........................grs. xxx.
M. et div. in pil. No. xx.

These are to be taken three or four times daily at first, subsequently to be reduced until only one is taken at bed-time. The treatment to be continued for three to nine months.—*Popular Science News.*

**A New Contrivance for Raising Patients.**—At the recent meeting of German naturalists and doctors, which was held at Frieburg-in-Bresgau from the 18th to the 22d of September, some interesting experiments were made in the surgical section with an apparatus which will, it is thought, be found very valuable in hospitals and in private houses where there are any sick persons. This is an apparatus placed by the bedside, and so contrived that, by using it, the most delicate person can raise a patient, no matter how heavy, from a recumbent position, change his linen, make his bed, and do all that may be required, without giving him the least pain. The inventor has put into execution the well-known principle for raising quarry stones by means of large claws which close through the weight of the object which they hold. In the apparatus referred to, the extremities of the claws are well wadded props which are inserted upon each side of the patient as he lies in bed. The patient finds himself stretched as in a hammock, and the weight of the body is uniformly distributed by means of a system of rollers adapted to the support of the apparatus. Bv a further contrivance the patient can be lifted into a bath placed beside the bed without any one touching him. It is said that all the doctors who witnessed the experiments made upon a sick person at Freiburg agreed that this apparatus was superior to anything hitherto invented for a similar purpose.—*Louisville Med. News.*

**Treatment of Premature Baldness.**—Dr. Lassar advances the theory (Berliner Klin. Wochenschrift) that premature baldness is the result of contagion, and not of any general condition of the health. The method of treatment which he suggests on this etiological basis is as follows: The scalp is to be washed every day with tar soap, or soft glycerine soap, or with soap containing sodium iodide; the soap is to be thoroughly applied, and rubbed into the scalp for fifteen minutes. Following this, a warm douche is used; then after the application of a corrosive sublimate solution (two parts in one thousand) the hair is dried, and a half per cent. spirited solution of naphthalin is rubbed into the affected parts. Carbolic or salicylic acid may be employed. If this treatment be adopted in the early stage, when the hair is just beginning to fall, it usually proves successful, but it must be kept up for eight weeks or more. The fact that this disease is due to a communicable morbid principle suggests that it may be conveyed by the comb and brush of the barber.—*Med. Age.*

**Micrococci** are now positively declared to be the cause of erysipelas by Dr. Fehleisen, Bergmann's assistant. He has isolated them, cultivated them on gelatine through fourteen generations. He then inoculated rabbits, and also men, with the pure organisms, and produced in most cases a typical erysipelas. The inoculations were made in seven patients who were suffering from lupus, cancer and sarcoma. One case of lupus was almost completely cured, in another case the cancerous tumors disappeared. In another of fibro-sarcoma the tumor diminished in size. In the other four cases no especial effect of the tumor was noticed.—*Med. Record.*

**The Treatment of Gonorrhœa.**—I believe that the reason of the usually tedious course of gonorrhœa is the too stimulating nature of the treatment so common in the early stages. If I can see the patient in twenty-four or thirty-six hours after the first signs of inflammation, I introduce an olive point double catheter attached to a Davidson's syringe. Through this force water of a commencing temperature of 70° or 80°, which increase as the patient can bear it. Use from four to six quarts. After using this quantity of water, you will find the inflammation nearly subsided. Then in three hours commence the following injection:

℞ Muriate hydrastine..............................gr. x,
Iodoform..............................gr. iij,
Glycerine..............................ʒ j,
Sassafras water—q. s. ad..............................℥ ij.
M. ft. sol. Sig. Inject 4 times a day. Shake.

If there is much acidity of the urine, or scalding and burning, give the following internally:

℞ Ext. kava, kava, fl..............................℥ ss,
Ext. Rhus. aromatic. fl..............................ʒ ij,
Balsam copaiba..............................℥ ss,
Tr. cubebs, q. s. ad..............................℥ ij.
M. Sig. One teaspoonful 4 times a day.

If I can get my patients to take good care of themselves, I very seldom fail to have them all right in eight or ten days. Let the readers of the Gazette try it and I am sure they will be well repaid for their trouble, both in the results and in the gratitude which the experienced patient will shower on them.—*L. C. Schutt, M. D., in Ther. Gaz.*

**Iodoform in Ophthalmic Practice.**—The following conclusions are drawn by E. Fischer, of Gratz:

1. Iodoform is well borne by the majority of patients.
2. It is the most effective agent against pannus scrofulosus and trachoma.
3. It renders excellent service as an antiseptic.
4. It hastens granulation and speedy regeneration of corneal epithelium.
5. In dacryocytitis and its consequent blennorrhœa it is not to be under-estimated.

Kazaurow (Wratsch, No. 42, 1882), also speaks in the highest terms of the drug. In several cases of extraction he was unfortunate enough to have escape of vitreous, with luxation of the lens and consequent iridocyclitis or phthisis bulbi. In two such cases, after using iodoform dressings there was not the slightest appearance of inflammatory reaction.—*N. Y. Med. Times.*

**Wm. Harvey's Rem^ins.**—The remains of Wm. Harvey, discoverer of the circnlation of the blood, 1616, were recently removed from the vault under Hempstead Church and placed in Harvey Chapel in a sarcophagus provided by the Royal College of Physicians.—*Ex.*

**Painting with Tincture of Iodine in Variola.**—In 1881, a patient entered the hospital of Konotop suffering from lumbar pains, and other symptoms indicative of the onset of variola. To satisfy the patient, Dr. Vetroff painted all of the lumbar region with the tincture of iodine. The next day, this region was entirely covered by a variolous eruption, while only two vesicles were found on the rest of the body. The progress of the disease was very benign.

Having observed this curious case, Dr. Bojinski-Bojko (Vratch, No. 1, 1883), during the development of an epidemic of variola in his district, commenced to paint with the tincture of iodine the anterior surface of the thighs of all the patients who presented the precursory symptoms of that disease. In the four cases so treated, the eruption confined itself strictly to the paited limits, and the prognosis was very favorable. Attempts failed to substitute sinapisms for the tincture of iodine.—*La France Medicale.*

**Injections into the Optic Nerve.**—Pfluger (Soc. d'Ophth. d'Heidelberg) had injected in dogs two or three drops of a saturated solution of fluorescine, directing it toward the center, partly in the trunk of the nerve under the arachnoid, and partly into the sheath under the dura mater. In about two minutes, both eyes showed a fluorescence of the retina, which persisted for five weeks. This effect cannot be produced by introducing the fluid directly ; it cannot be made to pass through the circulation, and requires at least 8 grammes to produce the result. A small quantity injected into the orbital cellular tissue gave no result. This proves that there is a direct communication between the two retinæ by the course of the optic nerve and of the chiasm, a fact confirmed by the experiments of Knies and Horner, who have in the same manner injected Prussian blue in cadavers, obtaining a coloration of the optic nerves of both eyes-—*La France Medicale.*

**Preserving Cadavers.**—The following is the preservative method used in the London dissecting rooms :

The bodis are injected with a solution of one pound of crystallized carbolic acid in half a gallon of glycerine and half a gallon of spirit. Each body is then sewn up in calico and put in a tank, and a solution consisting of glycerine one quart, water and spirit half a-gallon each, and common carbolic acid half a pint, poured over it—*Ex.*

**Constipation.**—As a remedy for chronic constipation one drop of the tincture of belladonna, U. S. P., in half a tumbler of water three times a day, has been represented to be efficient. It is not unlikely that the water is entitled to as much consideration as the drug, for constipation often results from a too sparing use of it.—*Exchange.*

**Snake-Bite.**—In two cases of snake-bite, Dr. George H. Carpenter (Medical News), reports recovery following local and internal use of the compound tincture of iodine, a single dose of ten or fifteen drops being given and poultices of raw onions being applied. The bites were by a copperhead snake.—*Med. Annals.*

**Eucalyptus in the Treatment of Acute Tonsillitis.**—For some time past I have been using eucalyptus in cases of quinsy with very gratifying results. Dilute one drachm of the fluid extract with one ounce of warm water, and use as a gargle or spray every twenty minutes. The water must be as warm as the patient can bear it.

It has been my good fortune to see all the cases so treated recover speedily, without suppuration. No other remedy was used, except in one instance, I prescribed quinine.

It has occurred to me that, owing to its antiseptic properties and its special action on the respiratory tract, eucalyptus would be an excellent local application in diphtheria, either used as above or to medicate vapor for inhalation.—*Atlantic Jour. of Med.*

**Salicylic Acid for Venereal Warts and Ulcers.**—Dr. Solon D. Stone has had very good results. His method is to fill, or pack an ulcer with the acid, which he keeps constantly applied until there is a healthy granulating surface. For a few minutes following the application of the acid to a raw surface the pain is quite severe, but it soon subsides.—*Med, and Surg. Rep., June 2.*

**Salicylate of Soda in Granular Conjunctivitis.**—A correspondent says that he has found that a solution of salicylate of soda, when applied to the everted lid in eases of granular conjunctivitis, will almost instantly relieve the pain following the use of sulphate of copper.—*Med. Review.*

**A Liniment for Rheumatism.**—The Quarterly Therapeutic Review says, methyl salicylate (oil of wintergreen) mixed with an equal quantity of olive oil or linimentum saponis, applied externally to inflamed joints affected by acute rheumatism, affords instant relief, and, having a pleasant odor, its use is very agreeable.—*Med. Age.*

**The Antiquity of the Trephine.**—In an editorial on this subject in the *Medical News* the following occurs: "The trephine dates back to, at least, five hundred years before Christ. No such instrument, however, existed in the Stone Age, and the openings are of such character as to forbid the surmise that any similar instrument was used. Most probably it was done by scraping, a method which, while tedious and painful in the adult, Broca has shown would be very brief, not exceeding four minutes, in the thinner and softer skull of childhood.—*Med. and Surg. Reporter.*

**Opium Poisoning**—*Nitrite of Amyl.*—Two cases are recorded by Dr. Turner, in the St. Louis Courier of Medicine, of recovery from opium poisoning under the use of nitrite of amyl Both cases were apparently bad ones, one of them being a child of six months, who had got half a grain of morphia by mistake. The inhalation was given very carefully, a few inspirations of the vapor at a time. The effect was apparent from the first, in the way of improvement of the pulse and respiration. After some hours the treatment was discontinued.—*Glasgow Med. Jour.*

